# Early Development of the GABAergic System and the Associated Risks of Neonatal Anesthesia

**DOI:** 10.3390/ijms222312951

**Published:** 2021-11-30

**Authors:** David A. Gascoigne, Natalya A. Serdyukova, Daniil P. Aksenov

**Affiliations:** 1 Department of Radiology, NorthShore University HealthSystem, Evanston, IL 60201, USA; dgascoigne@northshore.org; 2 Department of Biomedical Engineering, Northwestern University, Evanston, IL 60208, USA; natalya.serdyukova@northwestern.edu; 3 Department of Pediatrics, NorthShore University HealthSystem, Evanston, IL 60201, USA; 4 Department of Anesthesiology, NorthShore University HealthSystem, Evanston, IL 60201, USA

**Keywords:** interneurons, neurovascular coupling, excitatory/inhibitory balance, toxicity, MRI, animal, human, sevoflurane, isoflurane, infants

## Abstract

Human and animal studies have elucidated the apparent neurodevelopmental effects resulting from neonatal anesthesia. Observations of learning and behavioral deficits in children, who were exposed to anesthesia early in development, have instigated a flurry of studies that have predominantly utilized animal models to further interrogate the mechanisms of neonatal anesthesia-induced neurotoxicity. Specifically, while neonatal anesthesia has demonstrated its propensity to affect multiple cell types in the brain, it has shown to have a particularly detrimental effect on the gamma aminobutyric acid (GABA)ergic system, which contributes to the observed learning and behavioral deficits. The damage to GABAergic neurons, resulting from neonatal anesthesia, seems to involve structure-specific changes in excitatory-inhibitory balance and neurovascular coupling, which manifest following a significant interval after neonatal anesthesia exposure. Thus, to better understand how neonatal anesthesia affects the GABAergic system, we first review the early development of the GABAergic system in various structures that have been the focus of neonatal anesthesia research. This is followed by an explanation that, due to the prolonged developmental curve of the GABAergic system, the entirety of the negative effects of neonatal anesthesia on learning and behavior in children are not immediately evident, but instead take a substantial amount of time (years) to fully develop. In order to address these concerns going forward, we subsequently offer a variety of in vivo methods which can be used to record these delayed effects.

## 1. Introduction

Approximately 650,000 children born each year in the USA undergo a type of medical procedure that necessitates the administration of at least one dose of general anesthesia before their third birthday [1]. There is a growing concern regarding the potential for learning and behavior deficits in later childhood, as a result. This problem has garnered greater attention in recent decades in response to a series of retrospective studies, which identified an increased risk of learning and behavior deficits in later childhood following anesthesia exposure as a young child. For example, Wilder et al. [2] identified a significant relationship between anesthesia exposure before the age of four and math learning disabilities, and Chemaly and colleagues [3] found a significant relationship between anesthesia exposure and long-term abnormal behavior, where the likelihood of abnormal behavior increased with anesthesia duration but decreased as the child grew older. These notable studies highlight the potential consequences of neonatal anesthesia exposure, for which, several further studies and reviews have provided converging evidence [2,4,5,6,7,8]. Primarily, the specific learning and behavioral deficits that have been identified include, slower than normal reading, writing and arithmetic development, memory deficiency, abnormal social activity and the increased severity and frequency of disruptive behaviors. To investigate the mechanisms underlining neonatal anesthesia-related deficits, animal models have predominantly been employed.

Typical animal model studies adopted histological and immunohistochemical analyses and/or behavioral methodologies in rodents, to quantify differences at various points in development resulting from neonatal anesthesia exposure. A leading hypothesis of such studies proposed that anesthesia has neurotoxic effects that can lead to neuroapoptosis. This hypothesis has been largely supported by multiple studies which have identified elevated levels of neuroapoptosis in the thalamus, sensory cortex, hippocampus, prefrontal cortex, amygdala and cerebellum following neonatal anesthesia [9,10,11,12,13,14] (for a full review of affected structures, see [15]). Anesthesia toxicity has also been quantified in relation to dosage and duration. Intuitively, higher dosages and increased durations (e.g., a 6-hour (h) duration compared to a 2-h duration) engender more profound neurotoxic and neuroapoptotic effects [16]. Note, the duration of post-operative anesthesia recovery should be also taken into account.

Recent studies in children have further clarified the relationship between duration/frequency of anesthesia and developmental consequences. Three notable studies, the General Anesthesia Spinal (GAS) study, the Pediatric Anesthesia Neurodevelopment Assessment (PANDA) and the Mayo Anesthesia Safety in Kids (MASK) study, analyzed shorter durations (averaging close to 1 h for single doses) of anesthesia exposure and indicated the necessity for longer durations and/or multiple exposures to anesthesia in young children for later learning and behavioral deficits to be visible [17,18,19,20]. Additional support of this can be found in recent studies [21,22], which directly showed that the risk associated with neonatal anesthesia was more pronounced in children who were exposed at younger ages, underwent multiple exposures and experienced longer cumulative durations of anesthesia.

A key marker of the physiological consequences of neonatal anesthesia is neurotoxicity and neuroapoptosis. The neurotoxicity resulting from anesthesia is temporally limited to the duration of anesthesia and shortly thereafter while the agent is being expelled from the body; regarding inhalation agents, such as sevoflurane and isoflurane, the recovery of neuronal activity is relatively quick (e.g., 9 min after the end of one minimum alveolar concentration (MAC) sevoflurane anesthesia single neuron activity recovered to 63% of the awake level) [23]. Neuroapoptosis, on the other hand, has shown to continue developing for a few days past this period of toxicity [24]. These effects are known to impact a variety of cell types, including oligodendrocytes, astrocytes, pyramidal cells and interneurons, however, these cells are affected disproportionately [15]. This can induce developmental restructuring of local neuronal networks with a subsequent shift in the normal excitatory/inhibitory (E-I) balance of neuronal signaling in structures involved in learning and the control of behavior. A very recent study has found alterations in gamma oscillations, neuroplasticity and long-term potentiation (LTP), following general anesthesia in the main output from the hippocampus, the subiculum [25]. These results expand upon the findings of another study that demonstrated interneuron apoptosis and the downregulation of gamma-aminobutyric acid (GABA) producing enzymes in the cerebral cortex following neonatal anesthesia [26]. Evidently, the E-I network in these regions, which function via the use of the brain’s primary inhibitory neurotransmitter, GABA, was directly affected by the anesthesia. Thus, if neonatal anesthesia can significantly disturb local GABAergic functioning in certain structures, it could manifest as changes in learning and behavior.

Taking into account the importance of the GABAergic system in neuronal networks, we will briefly review the functions of this system and the critical period of its development, around the time of neonatal anesthesia exposure. This will provide context to the subsequent analysis of the neurotoxic and neuroapoptotic consequences of neonatal anesthesia during GABAergic development, and how this may be related to the learning and behavioral deficits observed following neonatal anesthesia exposure. Lastly, we will discuss current and prospective methodologies that can be applied in vivo to detect changes in the natural GABAergic functioning and development in children following neonatal anesthesia.

## 2. Function and Development of the GABAergic System

### 2.1. GABAergic Inhibition and Neurovascular Coupling

In the mature brain, the GABAergic system is fully capable of spatially and temporally confining neuronal excitation. Inhibitory neurons specialized for this task (interneurons) rapidly transmit GABA to GABA_A_ and GABA_B_ receptors located on neighboring cells. These transmembrane GABA-gated receptors facilitate the diffusion of chloride ions (Cl^−^) into the cells, creating a hyperpolarized state. This effect dramatically increases the input stimulus required for an action potential to occur, thus, inhibiting neuronal activity as a consequence [27]. Plainly, the inhibitory function of interneurons primarily helps to prevent the over excitation of neuronal networks, which could otherwise lead to seizures.

Various forms of GABAergic inhibition exist. Interneurons are known to create feedforward inhibition, which suppresses the activity of downstream neurons, as well as feedback inhibition, which projects inhibitory signals to upstream neurons. It has also long been established that interneurons are capable of disinhibiting other interneurons; although the functional purpose of this type of circuitry is still not fully understood [28]. Moreover, GABAergic terminals can make connections at almost any location along the target cell (e.g., axon initial segments, the perisomatic region and dendritic braches) [29,30], depending on the cell type and brain structure [31].

In addition to the traditional views on neuronal inhibition, GABAergic signaling is also involved in the regulation of cerebral blood flow. We would like to make a particular emphasis on this function because of its potential diagnostic and mechanistic value related to the long-term effects of neonatal anesthesia exposure [32].

Neurovascular coupling refers to the integrated relationship regarding how neurons in the brain and the surrounding vasculature interact to support focal increases in oxygen demand. This process is typically thought to result from increased neuronal activity, thereby leading studies to often focus on the excitatory system’s contribution to neurovascular coupling. Examples of the excitatory system’s involvement include glutamatergic and astrocytic pathways which utilize nitric oxide (NO), calcium ions (Ca^2+^), potassium ions (K^+^), and arachidonic acid metabolites to help produce local increases in cerebral blood flow ([33,34,35,36]) (for a review of how these components contribute to the neurovascular coupling, see [37]). However, recent reviews [34,38] have indicated that these mechanisms may not completely account for all aspects of neurovascular coupling. The authors report caveats, such as: NO is not the active signaling molecule in the cerebral cortex (though its presence is necessary for vasodilation to occur), Ca^2+^ increases from astrocyte projections are not necessary for vasodilation to occur and often take place following a significant latency period after the initiation of vasodilation and K^+^ siphoning via astrocytes does not always play a major part in neurovascular coupling. The separate facet of the fast-acting GABAergic system’s involvement in neurovascular coupling provides a more complete picture of how neurovascular coupling carries out its essential function. Thus, for the purpose of this review, we will focus on the GABA-dependent neurovascular interactions and its mechanistic role in the delayed side-effects of neonatal anesthesia.

Evidence has shown that GABAergic interneurons are involved in neurovascular coupling, as they are essential for the full expression of the hemodynamic response during various forms of stimulation (e.g., chemical [39], electrical [39], sensory [40] and optogenetic [41]) and epileptiform activity [42]. Support for these interactions can be seen in the fact that GABA receptors can be found along the surface of arterioles [43] where GABAergic interneurons make direct and functionally active morphological connections [28,44]. This suggests that these GABAergic projections induce the vasodilation of arterioles by-way-of hyperpolarizing the smooth muscle cells. The means of rapid signaling provided by GABAergic interneurons can enhance neurovascular coupling, particularly at the initial stage of hemodynamic response.

It is also important to note that inhibition has the added benefit of curtailing increases in oxygen demand. Urgent increases in oxygen demand can occur almost immediately when there is increased neuronal activity. GABAergic signaling can, therefore, have the dual effect of lowering oxygen demand, to remain within an accommodable range, and eliciting an increase in oxygen delivery via neurovascular coupling. This effect exists to prevent localized hypoxia. If the GABAergic system were to be debilitated or suppressed by any means, excitatory signaling would prevail and spread. In addition to the increased metabolic rate in such a case, due to the elevated frequency of action potentials, GABAergic facilitation of the hemodynamic response would also become compromised. Combined, these effects, of an increase in oxygen demand and a weakened hemodynamic response, can lead to hypoxic conditions [38] which may participate in the long-term side effects of neonatal anesthesia.

### 2.2. Structure-Specific GABAergic System Function

The GABAergic system is non-uniform throughout the central nervous system, and nearly each brain structure has its own specifically organized inhibition. Thus, to demonstrate this, we will focus on idiosyncrasies in structures that are both, well researched in relation to the consequences of neonatal anesthesia, and are either directly or indirectly involved in learning and behavior (i.e., the cerebral cortex, thalamus, hippocampus and cerebellum).

The cerebral cortex is often attributed as the structure where cognitive, perceptual and emotion processing take place [45]. The inhibitory operations in this region are carried out by local circuit GABAergic interneurons, which comprise approximately 20% of the neurons in the cerebral cortex [46]; the rest, primarily being glutamatergic excitatory neurons with both local and more distant synaptic targets.

The thalamus is widely accepted as a relay for sensory and motor signaling to and between cortical regions, but it is also an integral part of maintaining attention towards task-relevant stimuli [47]. The GABAergic system in the thalamus is comprised by local interneurons and neurons of the thalamic reticular nucleus (TRN) [48]. These neurons participate in the production of spontaneous and evoked (i.e., in response to stimulation) synchronous activity associated with movement, sleep and seizures [49].

In the hippocampus, interneurons comprise 10–15% of the total neuronal population, [50] play a central role in regulating both excitatory projections and synchronous activity [51,52] as well as facilitate neuroplasticity [53]. Research focused on the hippocampus has revealed its critical role in explicit memory. However, it is also important to note its integral contributions to other operations such as self-directed attention [54], emotional behavior [55], regulation of the hypothalamus [56], etc.

The anatomy of GABAergic signaling in the cerebellum is comprised of the Purkinje, basket, stellate and Golgi cells, where Purkinje cells [57] represent the only output from the cerebellar cortex and account for approximately 20% of cerebellar neurons [58]. Thus, Purkinje cells send inhibitory projections to deep cerebellar nuclei and are necessary for its characteristic functions—the generation of refined motor output and participation in motor learning [59].

In the cerebral cortex and hippocampus, where glutamatergic pyramidal cells vastly outnumber GABAergic interneurons [60], E-I balance can still be supported due to the fact that interneurons participate in synaptic connections with multiple excitatory neurons. This results in almost simultaneous post-synaptic inhibition of the pyramidal cells, both in terms of the time of initiation and the duration [61]. Interestingly, immediately after inhibition, simultaneously inhibited neurons have a high propensity to fire synchronously as well [60,62]. We should distinguish this type of local and physiological neuronal synchronization from long-lasting, widespread synchronization, which results from the failure of the inhibitory system (e.g., epileptic activity).

Physiological synchronized cortical activity is an essential part of cognition and perception. This type of neuronal synchronization allows excitatory signals to be distinct from the surrounding “noise” of the spontaneous baseline activity [61]. It has been well established that such synchronized activity (i.e., neurons firing within 10–20 ms of one another) can lead to long-term potentiation or depression of the involved synapses [63,64,65]. These lasting changes in neuronal circuitry represent a basis for the formation of memory and cognition at the cellular level; a notion that has been supported by an array of studies (for an in-depth review, see [66]). Thus, the GABAergic system not only regulates the activity of individual neurons and contributes to their metabolic function, but it is also an integral part of learning, memory, cognition and the resulting behavior.

### 2.3. Physiology of the E-I Balance

The E-I balance is a widely accepted term to indicate the proportions of excitatory and inhibitory inputs, which either emerge on a single neuron or exist within a neuronal network. Generally, E-I balance of a single neuron is viewed as a combination of two counteractive forces projecting to the neuron: excitation and inhibition. Simply, if the level of excitatory input increases, the firing rate of the neuron is also expected to increase, and if the level of inhibitory input increases, the firing rate of the neuron is expected to drop accordingly.

In cases of neuronal networks, this process is vastly more complex. A single excitatory neuron can make synaptic connections to multiple other neurons. Therefore, its projections have the capacity to activate numerous targets. This creates serial activation that will propagate until it is confined by inhibitory inputs, most often generated by interneurons. Since increased inhibition will expectedly result in a local or general decrease in responsiveness [67,68,69], such as is seen in the immediate and temporary effect of anesthesia, we will discuss more complicated shifts in the E-I balance towards excitation.

**Figure 1:**
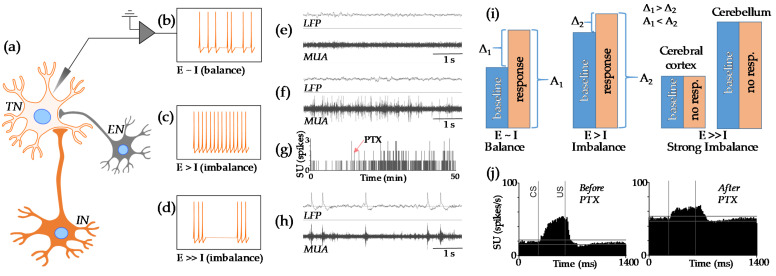
A schematic illustration of E-I physiology and balance. (**a**) A target neuron (TN) receives input from both inhibitory (IN) and excitatory neurons (EN). (**b**) If these inputs are “balanced”, TN fires relatively slowly. (**c**) If the inhibitory input is weak, the firing rate of TN increases. (**d**) If the inhibitory input is absent, TN can go into a seizure state. Additionally, we illustrated this scheme with examples of actual recordings. Local field potentials (LFP), multi-unit activity (MUA) and single units (SU) were recorded from layer IV of somatosensory cortex in awake adult rabbits (for experimental setup see [40]). (**e**) In the absence of stimulation, the baseline activity in somatosensory cortex can be very slow. (**f**) However, after the local injection of the gamma aminobutyric acid (GABA)-antagonist picrotoxin (PTX, 0.06 nmol/μL), neuronal activity visibly increased. (**g**) This effect is further shown in the temporal dynamics of spikes in a selected SU. Note that this neuron was nearly silent before injection. The red arrow indicates the time of the injection. (**h**) If the dose of PTX is higher (0.3 nmol/μL), both LFP and MUA go into seizure (functionally failed) states. (**i**) For these three scenarios the stimulus processing is characterized by a strong relative response to stimulation under a balanced state, by a weaker relative but higher in absolute magnitude response to stimulation under a slightly imbalanced state and by the absence of a response under a failed state (strong imbalance). Note that the nature of failed state depends on the brain structure. For example, in the cerebral cortex the failed state is related to seizure activity while in the cerebellum the failed state is related to the saturation of the neuronal response in highly elevated baseline. (**j**) Our actual data [70] illustrates the observation of a relatively small and strong imbalance (not shown). Activity of single units (N = 55) was recorded from deep cerebellar nuclei before and after injection of PTX (2.5 nmol/μL). PTX increased both baseline activity and the level of absolute response to conditioned (CS) and unconditioned (US) stimuli but decreased the relative (to the baseline) response.

Figure 1 shows the physiological basis for E-I balance (Figure 1a–d). Briefly, a small level of E-I imbalance results in the elevation of baseline activity. Consequently, this results in a decreased relative response and an increased magnitude in the absolute response (Figure 1e–g). If E-I imbalance is more severe, it will produce either seizures (Figure 1h) or the saturation of the neuronal response due to a highly elevated baseline (Figure 1i,j).

There is an ongoing discussion about the clinical manifestation of a mild E-I imbalance. For example, it has been suggested that a shift in the E-I balance towards excitation (under the state of reduced inhibition) could result in a reduction of the signal-to-noise ratio in neuronal circuits [71]. This would likely affect the efficiency of information processing [72], and it also represents one of the models for autism [71]. We would like to clarify this hypothesis. If the signal-to-noise ratio represents the relative neuronal response (indicated as “Δ” on Figure 1i) compared to baseline levels, the reduction in this ratio can be seen in the presence of increased baseline levels. However, we also would like to note that this is also accompanied by an adaptive increase in the absolute magnitude of response (indicated as “A” on Figure 1i). Thus, it is not clear what phenomenon would be responsible for the change in behavior: signal-to-noise ratio (“Δ”) or excessive absolute activation (“A”).

We must emphasize that if the level of E-I imbalance increases further, there will be a dissociation between electrophysiological states of different structures. For example, the cerebral cortex could go into a seizure state, whilst cerebellar neuronal responses may simply become non-visible due to the highly elevated neuronal baseline. The cerebral cortex seems to be very susceptible to seizures (Figure 1h) which can occur even after injections of a relatively low concentration of the GABA antagonist, picrotoxin (0.3 nmol/μL). Indeed, it has been concluded that unbalanced excitatory and inhibitory projections are the primary mechanisms of the transition from normal brain function to seizures, and that seizure-like activity can be reduced or even abolished by either increasing inhibition or decreasing excitation [73]. However, this observation is not true for all structures. For comparison, injections of a much higher concentration of picrotoxin (2.5 nmol/μL) into the cerebellum do not produce seizures, but merely elevate the baseline activity of neurons [70]. This discrepancy in the effect of reduced inhibition across different brain regions would therefore predict, if any of the structures were to exhibit a substantial change in E-I balance, it would manifest in a clinically visible and structurally specific manner.

### 2.4. GABA Signaling Development

Before the GABAergic system can function at the mature level, key developmental stages must first be reached. Such processes include the development of the GABA-driven force, GABAergic synapses and the spontaneous activity of GABAergic neurons.

Firstly, the GABA-driven force, which is the effect of GABA on postsynaptic currents, can transition from being excitatory to inhibitory over the course of development. An early indication that early GABA signaling can have an excitatory effect was reported in 1978, by Obata, Oide and Tanaka [74], and was later found to be as a result of GABA-induced depolarization by Ben Ari, in 1989 [75]. This effect has since been observed across a range of neurological structures such as, the hippocampus [76,77], neocortex [78,79], hypothalamus [80], cerebellum [81] and spinal cord [82,83], primarily through in vitro studies. The consistency between these findings across vertebrate species suggests this may be a universal phenomenon [84] and is often referred to as the excitatory to inhibitory switch.

The mechanism underlying the development of the GABA-driven force can be accounted for by changes in the direction of Cl^−^ diffusion. Immature GABAergic projections can increase the likelihood of an action potential by instigating an efflux of Cl^−^ which, rather than causing the characteristic hyperpolarization seen in adult cells [85], can incite depolarization. Both traditional optical fluorescence [86] and modified optical fluorescence [87] techniques, which measure the concentration of Cl^−^ within the cell, have been able to track the changing intracellular concentrations of Cl^−^ in immature cultured rat cells. These studies reported the transition from GABA-induced efflux to influx of Cl^−^ to occur between embryonic day 18 and postnatal day 14 [87] and between postnatal day 5 and 25 [86]. The foundation of this counterintuitive behavior of GABA signaling lies in the development of Cl^−^ transporters [84], which explains the observed accumulation and higher concentration of Cl^−^ within the immature neurons.

Broadly speaking, since observing the excitatory to inhibitory switch directly in children is not feasible, comparing studies that analyze the developmental timelines of the nervous system in rodents and humans allows us to make a reasonable prediction as to when it would occur. Comparable temporal changes in the cortical anisotropy were reported to occur around postnatal day 7 in rats and 40 weeks of gestation in humans [88], and other studies found the rapid myelination of white matter was seen through day 20 in rats [89] and 2 years of age in children [90]. This timeline comparison has been further supported by peak gliogenesis, axonal density and dendritic density occurring between postnatal day 7 and 10 in rats and at about 40 weeks of gestation in humans [88,91,92,93]. Additionally, the brain reaches 90% of its adult weight, and peaks in both synaptic development and myelination rate around postnatal day 20 in rats and 2 years of age in children [94,95,96,97] (for a full comparative review of neurological development between rodents and humans, see [98]). Therefore, while also considering some preliminary stages of GABA development have also been attained by 2.7 years of age in children [99], taken together, these findings indicate that the possible change in the GABA-driven force takes place within the first few years of life in children.

In addition to GABA-driven force development, GABAergic synapses develop and mature as well. A pioneering study that performed morphofunctional analysis was able to distinguish between GABAergic and glutamatergic synapses on hippocampal pyramidal cells [100]. The study concluded that synapses in 80% of neurons were functionally silent (i.e., did not produce evoked or spontaneous postsynaptic currents) at postnatal day 0 in rats. The synapses that were functionally active, exhibited postsynaptic currents induced exclusively by GABA-gated channels or both GABA and glutamate-gated channels. The same developmental sequence has also been identified in the developing primate hippocampus [101], and thus, is indicative of the propensity of GABAergic synapses to develop prior to glutamatergic synapses. Moreover, GABAergic synapses that have formed by postnatal day 12 in the rat hippocampus display high levels of synaptic plasticity, and GABA receptors that are located in silent synapses, can be activated via calcium currents [102]. In humans, the mature state of these synapses is not reached until the onset of puberty in the hippocampus and the end of adolescence in the neocortex [103]. Although, it is expected that the mature characteristics of these synapses will be achieved more rapidly in females, compared to males in certain brain regions [104]. Therefore, similarly to the development of the GABA-driven force, drastic GABAergic synaptic development may also commence during infancy, however, this process may last significantly longer.

The spontaneous activity of GABAergic interneurons can also take many years to develop in humans. The fast-spiking cortical interneurons, whose mature state is needed for higher cognitive functions [105,106], have shown to have lengthier developmental timelines than some of the aforementioned processes. For example, it was found that hippocampal basket cells, which have been suggested to be responsible for initiating gamma-band oscillations, exhibit notable alterations in their morphology as they transition from slow to high frequency cells between postnatal day 6 and 25 in mice [107], and a separate study noted that the adult electrophysiological properties of interneurons are not attained until some point ranging from postnatal day 7 to 40 in mice [108]. In line with this trajectory of interneuron development, the number of GABAergic release sites also increases through postnatal day 30 in the mouse cerebral cortex [109]. These reports suggest that the spontaneous frequency of mature interneurons is not fully acquired until much later in development (i.e., around late childhood and into adolescence), compared to both the GABA-driven force and activation of GABAergic synapses which likely develop considerably earlier.

### 2.5. Structure-Specific GABAergic Development

Since there are morphological distinctions between GABAergic structures, it is natural that there be developmental discrepancies as well. In order to maintain continuity, the structural development of the GABAergic system outlined below will be restricted to the brain regions that were previously discussed—the hippocampus, thalamus, cerebral cortex and cerebellum.

**Figure 2:**
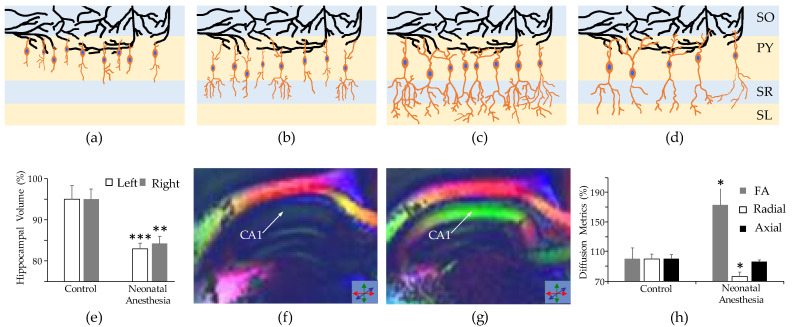
The impact of neonatal anesthesia on the development of interneurons in the hippocampus. (**a**–**c**) The schematic shows normal development of gamma aminobutyric acid (GABA)ergic interneurons (orange cell bodies with blue nuclei) in layers of the hippocampus. As the interneurons develop, (**a**) corresponds to the newborn state whereas (**c**) corresponds to the mature level of development), their axons lengthen and dendritic branches increase in complexity. Note, dendrites of target neurons (in black) are unchanged in the figures to demonstrate continuity of location in the observed neurons; in reality, they would also show morphological development concurrently with the interneurons. (**d**) Following exposure to neonatal anesthesia, we expect to see a decrease in the number of interneurons and reduction in their dendritic complexity. To compensate for the decrease in their quantity, interneurons can increase axonal length and number of targets. The following were adapted from our previous study [113]. (**e**–**h**) The results of a comparative analysis between control subjects and those exposed to neonatal anesthesia are shown. (**e**) Volumetry of the adult rabbit hippocampus revealed a significant reduction in the hippocampal volumes of subjects exposed to neonatal anesthesia. (**f**,**g**) Fractional anisotropy (FA) maps of representative adult subjects from the control (**f**) and neonatal anesthesia (**g**) groups show significant changes in the direction of water diffusion, with the most notable changes in the CA1 region (indicated by the arrows) (**h**), which exhibited a significant increase in FA and decrease in radial diffusivity, indicative of lower levels dendritic branching and neurodegeneration (represented in (**d**)). The layers of the hippocampus shown in (**a**–**d**) are labeled as follows: stratum oriens (SO), pyramidal cell (PY), stratum radiatum (SR) and stratum lacunosum-moleculare (SL) layer. (**e**,**h**) Statistical significance is indicated by the asterisks: one asterisk indicates *p* < 0.05, two asterisks indicate *p* < 0.01 and three asterisks indicate *p* < 0.001.

The hippocampus experiences rapid development in the postnatal period (Figure 2a–c). It has been shown that 20% of the dentate gyrus cells are yet to form following gestation [110]. That being said, interneurons in the hippocampus can exhibit mature physiological activity far earlier than pyramidal cells. This has been elucidated by a study which used patch clamp recordings on a sample of interneurons and pyramidal cells from the rat hippocampus at embryonic day 18 [111]. The study determined 65% of interneurons were functionally active while only 12% of pyramidal cells showed spontaneous and/or evoked potentials, thereby suggesting early hippocampal interneuron networks as the driving force of synapse development between the hippocampus and cerebral cortex [112].

Two distinct origins of GABAergic interneurons have been identified in the cerebral cortex. These lineages can be separated by their respective expression of transcription factors; 65% of such interneurons emanate from the neocortical ventricular and subventricular zone of the dorsal forebrain, with the rest (35%) originating from the ventral forebrain [114,115]. These interneurons experience a large wave of generation and migration between 10 to 25 weeks of gestation in humans and continue to develop postnatally in both rodents and humans [116,117]. The fully mature state of neurons in the prefrontal cortex can take as long as 25 years in humans to be reached [118].

Thalamic interneurons for the most part have an early midbrain ontogeny [119]. While the majority of neurogenesis and differentiation in the thalamus occurs during gestation [120,121], thalamocortical connectivity continues to develop during infancy, the saliency of which has been correlated to visual working memory performance and measures of learning ability [122]. It has been generally thought that there are local GABAergic connections between TRN neurons, however, recent evidence suggests that these connections exist only for the first two weeks in mice [123].

The peculiar GABAergic composition and function in the cerebellum, corresponds to a similarly specific developmental timeline. For example, GABAergic neurons of the rodent cerebellum have been shown to migrate radially from a single origin, the embryonic cerebellar primordium, during late gestation and early postnatal period [124]. Once the majority of the GABAergic neurons have migrated, the GABAergic synapses predominantly form during the second and third postnatal weeks in mice [57]. It is therefore possible that these changes likely occur in response to external environmental stimulation, as this is the same time period during which intensive motor learning is taking place.

While GABAergic neurons may follow a unique developmental process, for the most part, it is clear that the predominant structural development of the GABAergic system continues into early childhood. The different developmental timelines and sequences illustrate the respective periods where each structure may have an increased susceptibility to anesthesia-induced neurotoxicity.

## 3. Consequences of Neonatal Anesthesia

### 3.1. Neuroapoptosis in GABAergic Structures

Exposure to neonatal anesthesia can produce neurotoxic and neuroapoptotic effects in a variety of brain structures. To date, primarily animal models have been used to investigate the pathological effects of neonatal anesthesia. Reasons for this include allowing for comparable control groups, the standardization of methods and histological analysis of the affected tissues, all of which are less feasible in human studies. As such, reports using animal models will represent the majority of findings presented below.

In response to the learning and behavior deficits observed in children after neonatal anesthesia, the brain regions involved in such functions have been some of the most studied. For example, the hippocampus and hippocampus-related structures have been of great interest to researchers studying the pathological effects of neonatal anesthesia. Exposure to neonatal anesthesia has been shown to result in significant neuroapoptosis and a reduction in dendritic complexity in the hippocampus (Figure 2d). Maloney et al. [9] observed neuroapoptosis throughout the mouse hippocampus after 3 exposures to anesthesia, each two days apart for 6 h in total. The apoptosis observed by Jiang et al. [125], who also administered anesthesia for six cumulative hours, across three sessions on postnatal days 7, 14 and 21, was so severe that there was a significant reduction in the rat hippocampal volume, compared to what was observed in the control group. Similarly, a reduction in hippocampal volume has also been observed in adult rabbits after they were exposed to three instances of neonatal anesthesia [113] (Figure 2e). The same study also conducted diffusion tensor imaging and identified significant changes in the fractional anisotropy of the CA1 region (Figure 2f–h), indicative of a reduction in cells and dendritic branching. The neurotoxic effects of anesthesia have been shown to induce apoptosis to varying degrees across cell types in the hippocampus, with GABAergic neurons representing a proportion of apoptotic cells that is nearly four times larger than that of glutamatergic neurons [126].

In the cerebral cortex, a single dose of anesthesia, of three or more hours on postnatal day 6, has shown to induce notable apoptosis in the Macaque prefrontal cortex [12]. In addition, another study has reported a loss of 2% of total neurons in the mouse cerebral cortex, the majority of which were GABAergic neurons, and a dramatic decrease in *GAD65* and *GAD67* expression, following a single dose of anesthesia for 6 h [26].

In terms of the pathogenic effects of anesthesia in the cerebellum, significant neuroapoptosis has been observed in the granule layer [127], and Purkinje cells exhibit a reduction in density, connectivity and dendritic length [128]. The timing of these observed consequences of anesthesia may slightly precede and perhaps even coincide with the rapid GABAergic synaptic developmental period as these findings were reported between postnatal day 7 and 10 in mice.

Thalamic apoptosis from neonatal anesthesia has also been widely reported [9,10,11]. For example, one study revealed neuroapoptosis and behavioral deficits following neonatal anesthesia in the mouse thalamus; the effects of which, were worsened by repeated exposures [9]. A possible link between the neuroapoptosis and behavior deficits may be derived from the findings of another study, which found a single 6 h dose of anesthesia to induce alterations in excitatory and inhibitory transmission [129]. These results indicate that neurotoxicity and neuroapoptosis can induce changes in the E-I relationship that is necessary for the typical sensory and learning functioning of neuronal circuitry in the thalamus.

### 3.2. Short-Term Neurotoxicity

There are multiple possible mechanisms by which these observed changes may be explained. It has been suggested that anesthesia may elicit epigenetic changes resulting in the downregulation of the brain-derived neurotrophic factor due to the increased interaction among transcription factors [130], and/or the downregulation of GABA-synthesizing enzymes (*GAD65* and *GAD67*) [26]. Anesthesia may also be responsible for increased intracellular calcium levels from either an altered endoplasmic reticulum [131] or from increased glutamate signaling [132], which is known to induce neurodegeneration [133].

Since supplemental oxygen is often administered along with anesthetics (at concentrations ranging from 30–100%), anesthesia-related hyperoxia may also be a contributing factor. Hyperoxia can result in oxidative stress, where the accumulation of reactive oxygen species (ROS) resulting from hyperoxic conditions can oversaturate the natural antioxidant capacity of the brain and, therefore, lead to neurotoxicity [134]. This effect can be exacerbated if one were to combine general anesthesia with higher levels of supplemental oxygen. For example, isoflurane at 1MAC in 80% oxygen can create up to a 300% increase in the partial pressure of oxygen in brain tissue [23]. As demonstrated, a multitude of direct neurotoxic pathways may explain the neuroapoptosis observed immediately following neonatal anesthesia. However, as it remains, no clear consensus has been reached as to how much each pathway may contribute to neurotoxicity and the resultant neuroapoptosis, thus, highlighting the need for further interrogation.

The outcome of these mechanisms may not be universal, as it should also be noted that some sex differences have been identified in how neonatal anesthesia affects the GABAergic system. One such difference was identified by Aligny et al. [135] who observed anesthesia to substantially upregulate Cl^−^ transporters in the cortex of male rodents compared to male controls, while the anesthesia-exposed female Cl^−^ transporter levels to be indistinguishable from those of female controls. Another example can be seen in a study by Chung et al. [136], who observed reduced spontaneous inhibitory postsynaptic currents. These findings, together with the different rate of GABAergic development in females and males (note that this process can be region-specific), indicate a possible basis for subtle sex differences in the later developmental effects of neonatal anesthesia, such that females are more likely to exhibit impaired spatial learning and memory, as opposed to the tendency of males to present with non-spatial learning and memory deficits as well as anxious behavior (for a recent review of sex differences following neonatal anesthesia, see [137]).

### 3.3. Long-Term Neurovascular Coupling Deficiency

The role of GABAergic neurons in normal neurovascular interactions was reviewed in previous sections. However, since neonatal anesthesia affects the development of the GABAergic system, it can result in weakened hemodynamic functioning. Neurovascular deficiency in adult animals resulting from neonatal anesthesia has already been shown by changes in both blood oxygen level-dependent (BOLD) functional magnetic resonance imaging (fMRI), during stimulation [113,138] and regional resting state fMRI signals [139]. The observed reduction in the magnitude of BOLD fMRI response indicates that the deficiency is at the neuronal level, vascular level or both. The change in amplitude of low-frequency fluctuations in regional resting state fMRI illustrates that arteriolar vasomotion did not reach normal adult levels in subjects exposed to neonatal anesthesia [23,140,141]. The presence of neurovascular deficiency after neonatal anesthesia exposure raises an important question about the possibility of the delayed development of highly localized hypoxia, which, as previously reviewed, involves GABAergic functioning.

Generally, insufficient oxygen delivery, to meet the concurrent oxygen demand, can engender hypoxia. The pathological effects of this state are well known. Without a large enough oxygen supply to support neuronal activity, cell dysfunction or even death will inevitably transpire. In acute instances of hypoxia, such as ischemic strokes where there is a nearly entire cessation of oxygen delivery, necrosis from energetic failure occurs [142,143]. However, elevated levels of neuronal apoptosis are still present in less severe but chronic cases of hypoxia [144,145]. For chronic hypoxia to manifest the brain tissue oxygen should reach a specific “threshold” level when the biochemical properties of brain tissue start to change [38]. The formation of ROS is a typical result of cell metabolism. ROS play important physiological roles, such as in cell proliferation, differentiation and migration, when their concentrations are maintained within an acceptable range [146]. However, hypoxic conditions have been shown to upregulate the production of ROS beyond the intrinsic capacity of anti-oxidative systems, thereby inducing oxidative stress [146,147]. Oxidative stress is particularly detrimental to the health of cells, as there is a relatively high likelihood for the ROS to react with essential macromolecules (i.e., proteins, lipids, nucleic acids, membranes, etc.) and subsequently trigger apoptosis [148,149]. In addition to the accumulation of ROS, hypoxia can also cause a reduction in intracellular and extracellular pH [150,151], phosphocreatine concentration [151] and inorganic phosphate concentration [151,152] and an increase in the level of nicotinamide adenine dinucleotide (NADH) [151,153]. Each of these changes to the intracellular and extracellular composition can chronically debilitate the functions of neuronal networks and even lead to apoptosis. As illustrated, hypoxia can cause cell death via multiple mechanistic pathways, thus, cementing the importance of normal neurovascular coupling for heathy brain functioning and development.

The GABAergic system, as previously discussed, is particularly susceptible to neonatal anesthesia. The death of GABAergic interneurons would therefore not only cause a restructuring of local neuronal network, but also affect the GABAergic systems involvement in neurovascular coupling. Comparing neuroapoptosis from the expected anesthesia-related neurotoxicity and localized hypoxia due to neurovascular coupling deficiency, we should emphasize that the latter can have a delayed and progressive effect, which lasts weeks/months in the case of animal models and years in humans. Thus, this effect predicts that if neonatal anesthesia is delivered during a critical period of GABAergic development, the initial anesthesia-induced apoptosis will change local neuronal networks and this will later result in disseminated neuronal dysfunction, driven by highly localized areas of hypoxia.

Since neurovascular deficiency develops much later after neonatal anesthesia exposure, it raises the question of how it can be entangled with neurodegenerative diseases. The GABAergic system typically operates through Cl^−^ channels and, thus, it is important to acknowledge that there are several neurodegenerative diseases (e.g., Alzheimer’s, Parkinson’s and Huntington’s disease) which are associated with the dysfunction of these channels [38]. More specifically, selected studies have reported increased amyloid-β protein levels in response to neonatal anesthesia [154,155,156]. Therefore, there may be a link between the development and progression of neurodegenerative disease and neonatal anesthesia. In order to assert the validity of these possibilities and refine our understanding of anesthesia’s participation in the development of abnormalities throughout the lifespan, further interrogation is, indeed, required.

These possible mechanisms of neurotoxicity and neurovascular coupling deficiency recapitulate the striking consequences of neonatal anesthesia. Given that these changes occur during essential GABAergic development, they suggest impaired or altered GABAergic function to be a contributing factor to the learning and behavioral deficits seen in children. The physiological means by which anesthesia-induced dysfunctional GABAergic signaling can lead to such impairments can be elucidated by the apparent changes in the E-I balance.

### 3.4. E-I Imbalance after Neonatal Anesthesia

Since there are a range of possible severities of E-I imbalance, such that smaller shifts in this balance produce mostly disorders at the behavioral and cognitive level, whereas larger shifts result in neurological symptoms (e.g., seizures), we should first establish the typical level of imbalance accounted for by the delayed effects of neonatal anesthesia. There is a general consensus that profound neurological deficits (for example, epilepsy and cerebellar ataxia which manifest in cases of larger imbalances) are not typically associated with neonatal anesthesia [32,113,157]. Therefore, we will mostly discuss mild levels of E-I imbalance.

Unlike neurological deficits, the presence of diagnosable psychiatric disorders after neonatal anesthesia exposure is a controversial topic. In the case of autism, for example, it remains disputed whether or not it is linked to neonatal anesthesia [158,159]. On the other hand, learning deficiency is viewed as a primary sign of delayed complications after neonatal anesthesia [2]. It has been shown that the E-I balance in the prefrontal cortex is critical for working memory and associative learning [160], and that E-I balance in the cerebellum is needed for the expression of some classically conditioned responses [70,161].

There is a very limited number of studies that have evaluated E-I balance in adults following neonatal anesthesia exposure. However, one such study reported a change in GABA levels in adult rabbits who were exposed to repeated isoflurane anesthesia as neonates [113]. Some of these changes are associated with learning. For example, this study observed that lower GABA levels in the dentate gyrus of rabbits corresponded to lower GABAergic inhibition after normal learning, yet, such adaptations were not present in the adults who were exposed to neonatal anesthesia. Moreover, another study reported that the activity of local inhibitory interneuron networks was altered in adult mice after repeated neonatal exposure to propofol [162]. The authors found that parvalbumin-expressing and somatostatin-expressing interneurons were hypoactive, but vasoactive intestinal peptide-expressing interneurons were hyperactive when the mice performed a motor learning task. An in vitro study showed that neonatal isoflurane anesthesia exposure increased evoked excitatory postsynaptic currents (eEPSCs) twofold by means of α-amino-3-hydroxy-5-methyl-4-isoxazolepropionic acid (AMPA)-mediated mechanisms, and the amplitudes of evoked inhibitory postsynaptic currents (eIPSCs) were also increased in the adolescent rat thalamus [129]. These findings indicate that neonatal anesthesia can lead to long-lasting alterations in the excitability of neurons, where the increased magnitude of eIPSCs may be a specific adaptation to compensate for the existing E-I imbalance.

Taken together, these data illustrate that the level of E-I imbalance after neonatal anesthesia is generally not sufficient to induce clear neurological or psychiatric disorders, but that it may indeed be enough to produce learning/memory deficiencies.

## 4. Methodological Outlook

In this section, we will review in vivo methods which can assess the natural role of GABA and E-I balance in neuronal dysfunction following neonatal anesthesia exposure in humans and animals. Generally, this type of assessment is expected to be a technically difficult task, because it should be done in anesthesia-, stress- and pain-free subjects, as these confounds can cause neuronal synchronization, among other effects [30,148,149,150,151], which would artificially alter GABAergic signaling and the observed E-I balance.

### 4.1. Neuroimaging Techniques

Magnetic Resonance (MR) neuroimaging studies may prove to be vital tools to investigate the development of the GABAergic system going forward in both humans and animals. The most obvious choice is MR Spectroscopy (MRS) which has the ability to detect endogenous metabolites in vivo [163]. Since GABA concentrations in the brain in general are relatively small, and the natural behavior of GABA in the magnetic field makes the precise detection of GABA difficult, MRS would have to employ spectral editing methods in high-power imagers (i.e., 3 Tesla and above) [164]. With these adjustments, MRS can then be used to measure changes in GABA activity [165,166,167] after neonatal anesthesia. The very first data in this area of research has only been recently acquired [113]. While MRS shows strong promise for future research, the spatial resolution of clinical MRS may not be sufficient to detect the small and disseminated alterations in the GABAergic system.

BOLD fMRI is a method based on the dynamics of oxy/deoxyhemoglobin [168] and, thus, can indirectly assess oxygen delivery via the hemodynamic signal and neurovascular interactions [169]. BOLD fMRI has already shown to be effective in identifying changes in the hemodynamic response of adult rabbits who were exposed to neonatal anesthesia [113,138], and it, furthermore, has the potential to investigate E-I balance in awake stress-free animals before and after local injection of a pharmacological agents [40]. Even though, delayed anesthesia-induced changes in the hemodynamic response may be small, BOLD fMRI appears to have the capacity to identify such changes provided the imaging is structurally targeted. Contrast imaging can further increase the specificity of these MR approaches [170], however, this method may be hazardous to children due to the potential toxicity of the contrast agents. On the other hand, resting state fMRI, which aims to draw conclusions about the functional connectivity of brain regions, has already proven its capacity to detect changes in the developing neonatal hippocampus [171]. Moreover, regional resting state fMRI has also been used to observe changes in hemodynamic signals after neonatal anesthesia [139]. A separate MR modality, Diffusion Tensor Imaging, is capable of tracking white matter maturation processes across development [172]. This technique measures the magnitude and direction of water diffusion [173], and is sensitive to factors that affect tissue anisotropy, such as axonal density or dendritic arborization [113]. Although these indicators do not provide direct information about GABA function, they can be useful in the assessing changes in axons and dendrites in selected structures.

Additional (non-MR based) neuroimaging methodologies include volumetry techniques such as computed tomography (CT) and positron emission tomography scanning (PET). These methods would prove useful for detecting developmental abnormalities in brain structure in cases where MR modalities would not be appropriate. Although helical CT scans with a contrast agent can create a 3D visualization of blood vessels in the brain [174], further technological advancements may be required to map the architecture of the microvasculature.

### 4.2. Neuroimaging Alternatives

In children, some alternatives to neuroimaging have the ability to provide better temporal resolution. Primarily, electroencephalography (EEG) and magnetoencephalography (MEG) are non-invasive methods of measuring cortical neuronal activity [175]. EEG measures changes in the brain electrical field, while MEG records changes in the magnetic field produced by neuronal activity. These methods have often been used to understand the physiological basis of cognition in the cortex [176,177], however, they may also be able to detect physiological changes in the cortex following neonatal anesthesia. Although, yet again, we must stress the consideration that the spatial acuity required to observe the physiological consequences of neonatal anesthesia may first necessitate that further technological advancements be made to record brain activity non-invasively.

Animal models, on the other hand, provide more methodological flexibility. Appropriate control groups can be used as well as invasive approaches to investigate changes in structural function. All of the above methodologies are suitable for use in animal models, but the choice of which to use should depend on the susceptibility of the awake animal to stress.

In animals, localized laser Doppler techniques can assess relative changes in cerebral blood flow to help investigate neurovascular coupling in vivo [178], and two-photon microscopy can represent neuronal structure in three dimensions with concurrent recording of both vasomotion and neuronal activity [179]. Moreover, classical recording of single neurons in vivo with implanted electrodes can also provide information about activity of both inhibitory and excitatory cells [180,181], and direct, localized brain tissue oxygen recordings with microelectrodes [23] can have the added benefit of evaluating oxygen dynamics which depend on GABAergic involvement in neurovascular coupling.

In the immediate future, we anticipate these methods will be some of the most prolific in studying the basic mechanics of how neonatal anesthesia affects both GABAergic development and neurovascular interactions.

## 5. Concluding Remarks

After describing the primary role of the GABAergic system and how it develops to achieve its functions, we outlined neonatal anesthesia’s disruptive role in these processes. As illustrated the GABAergic system is essential in a range of processes from maintaining E-I balance to its dual role in both regulating the rate of oxygen consumption and supporting oxygen delivery. The disproportionate neurotoxic and neuroapoptotic effects of neonatal anesthesia can negatively affect the GABAergic system in structures associated with learning and behavior, during their critical periods of development. Therefore, we conclude, neonatal anesthesia’s effect on GABAergic development can account for some of the delayed learning and behavioral abnormalities seen in older children.

Animal models have definitively concluded that neuroapoptosis is evident throughout the brain following neonatal anesthesia exposure in animals. The majority of human cohort studies which show conflicting results, that neonatal anesthesia in children may not be as dangerous as animal models suggest, have drawn such conclusions based on much shorter durations of anesthesia exposure. Animal models often administer multiple doses for around six cumulative hours in duration, while the observational reports in children have mostly been restricted to a single dose of anesthesia for approximately 1 h. While, indeed, a single, short duration of neonatal anesthesia may be relatively harmless in children, the risk of later deficits increases with duration, frequency and the earlier in development the anesthesia is administered [20]. Moreover, the evaluation of the neurotoxic effects of neonatal anesthesia in children is difficult, because it has still not been established at what age the learning deficiency will start to manifest.

We suggest that the negative effects of neonatal anesthesia on learning and behavior will not be immediate and will need a substantial amount of time (years) to fully develop. This delay can be due to the prolonged developmental curve of the GABAergic system. It appears that the impact of neonatal anesthesia on behavior takes time to develop [6] and can become more visible once the patient approaches adolescence. Thus, we do not anticipate the direct consequences of neonatal anesthesia to last perpetually and continue affecting neurons into young adulthood, although the learning/behavioral changes that occur, as a result, will persist. However, the exact timing of when the developmental effects first become clinically visible, remains to be illuminated through the use of longitudinal studies with frequent assessment throughout their duration.

When general anesthetics are administered to infants for long single or cumulative durations, the risk of developmental abnormalities must be considered. This temporal confinement can be seen by the gradual reduction in anesthesia-dependent neuroapoptotic dynamics which starts around postnatal day 14 in rodents [182], and retrospective analyses, focused on children and adolescents who had been exposed to anesthesia early in development, which indicate that the greatest vulnerability of developmental consequences is until approximately 4 years of age [2,22,183]. Thus, with the support of future research we recommend additional considerations be made when deciding the most appropriate course of treatment on a case-by-case basis, such as, the use of regional anesthesia over general anesthesia, possibly delaying intervention until later in development, and the administration of supplemental oxygen under monitoring by non-invasive near-infrared spectroscopy (NIRS)-based cerebral oximetry.

To address these concerns going forward, we have reviewed the neuroimaging and non-neuroimaging techniques that can be utilized in vivo to evaluate the delayed effects of neonatal anesthesia. Studies in humans will likely rely on versatile MR methodologies as they are widely applicable and are undergoing continual development. However, MEG and EEG may also be needed to strengthen MRI findings. Although these techniques will eventually prove useful in humans, neuroimaging and non-neuroimaging techniques that can be used in animal models show the most immediate promise. Animal models will be able to elucidate the connections between the immediate and delayed neurotoxic and neuroapoptotic consequences of anesthesia and the functional, instrumental analysis conducted later in development.

## Data Availability

The data presented in this study are available on request from the corresponding author.
